# Lectin of Concanavalin A as an anti-hepatoma therapeutic agent

**DOI:** 10.1186/1423-0127-16-10

**Published:** 2009-01-19

**Authors:** Huan-Yao Lei, Chih-Peng Chang

**Affiliations:** 1 Department of Microbiology & Immunology, and Institute of Basic Medical Sciences, College of Medicine, National Cheng Kung University, Tainan, Taiwan

## Abstract

Liver cancer is the predominant cause of cancer mortality in males of Southern China and Taiwan. The current therapy is not satisfactory, and more effective treatments are needed. In the search for new therapies for liver tumor, we found that Concanavalin A (Con A), a lectin from Jack bean seeds, can have a potent anti-hepatoma effect. Con A after binding to the mannose moiety on the cell membrane glycoprotein is internalized preferentially to the mitochondria. An autophagy is triggered which leads to cell death. Con A as a T cell mitogen subsequently activates the immune response in the liver and results in the eradication of the tumor in a murine *in situ *hepatoma model. The liver tumor nodule formation is inhibited by the CD8^+ ^T cells, and a tumor antigen-specific immune memory is established during the hepatic inflammation. The dual properties (autophagic cytotoxicity and immunomodulation) via the specific carbohydrate binding let Con A exert a potent anti-hepatoma therapeutic effect. The novel mechanism of the Con A anti-hepatoma effect is discussed. The prototype of Con with an anti-hepatoma activity gives support to the search for other natural lectins as anti-cancer compounds.

## Review

### Liver cancer and hepatocarcinogenesis

Liver cancer is the fifth most important cancer worldwide, and is the third most common cause of cancer mortality because of the very poor prognosis [1]. Most liver cancers are hepatocellular carcinomas (HCC) which have unique epidemiologic features with dynamic temporal trends, variations in different geographic regions, racial and ethnic group, sex, and risk factors. The highest incidence rates are in Africa and eastern Asia. China alone accounts for more than 50% of the world's cases. Incidence is low in most developed countries, except for Japan, while a moderately increased incidence occurs in southern European countries. The major risk factors for HCC are chronic infection with the hepatitis B (HBV) and C (HCV) virus, although aflatoxins are thought to be an environmental factor in tropical areas due to the contamination of food with fungus. In Asia, the dominant risk factor is chronic HBV infection that is largely acquired by maternal-child transmission. However, in Japan or in Western countries, the dominant hepatitis virus is HCV [2,3]. Individuals who are chronic carriers of HBV have a greater than 100-fold increased relative risk of developing a tumor. The universal immunization of infants with the HBsAg vaccine not only decreases the HBV carrier rate, but also reduces the incidence and mortality rate from HCC in Taiwanese children [4]. Chronic active hepatitis is recognized as the major risk factor for HCC, and is accompanied by liver cell necrosis, inflammation, cytokine abnormal synthesis and fibrosis. In Asia, America and Europe, 90% of HCC cases are associated with cirrhosis. Three steps (hepatitis, cirrhosis, hepatocarcinogenesis) are involved in the evolution of HCC tumor formation post HBV/HCV chronic infection [5].

The most common condition associated with hepatocarcinogenesis is cirrhosis, which develops after a latency of 20–40 years of chronic liver disease. HCC risk remains low during chronic liver disease but exponentially increases at the cirrhosis stage, suggesting that important events precipitate the increase in liver tumor formation at the cirrhosis stage. Liver cell proliferation is increased during chronic hepatitis, but cirrhosis is characterized by decreasing hepatocyte proliferation, indicating that the regenerative capacity of the liver is exhausted at the cirrhosis stage. Both cell-intrinsic and cell-extrinsic alterations are responsible for the cirrhosis-associated hepatocarcinogenesis [5]. Telomere shorting is suggested to explain the limited regenerative reserve of liver cells. Telomere shorting not only leads to an activation of the cell cycle and apoptosis checkpoints that restrain the proliferative capacity of liver cells and in turn select for hepatocytes carrying deletions of checkpoint genes, but also induces chromosomal instability, which can accelerate the further loss of the checkpoint function. Cirrhosis also causes alterations of the liver environment by altering the cytokine secretion from activated stellate cells as well as inflammatory signaling from infiltrating immune cells. Furthermore, the decrease in liver function at the cirrhosis stage could increase toxic metabolites in the blood serum, inducing alteration of the macroenvironment. The altered milieu of both microenvironment (local factors in the liver) and the macroenvironment (systemic acting factors) could stimulate the impaired hepatocyte proliferation at the cirrhosis stage, leading to a further selection of genetically altered pre-malignant clone of transformed hepatocytes and then tumor formation.

### Abnormal glycosylation on tumor cells and lectins

Glycoconjugates, consisting of large glycoproteins, play an important protective role in many biological phenomena [6]. The number of complex N-glycan and degree of branching cooperate to regulate cell proliferation and differentiation. Growth factor receptors have high numbers of N-glycans and, therefore high avidities for the galectin lattice. The surface levels of glycoprotein expression will determine the transition between cell growth and arrest [7,8]. An altered glycosylation of functionally important membrane glycoproteins would affect tumor cell adhesion or motility, resulting in invasion and metastasis. For example, aberrant sialylation in cancer cells is found to associate with malignant properties of invasiveness and metastasis. Carbohydrate antigen sialyl Lewis a (CA19-9) is used as a serum tumor marker for diagnosis of cancers in the digestive organs. A high expression of sialoglycoconjugates in colorectal cancer is significantly associated with poor prognosis and lymph node metastasis [9].

Lectins were previously used to define agglutinins that could discriminate among types of red blood cells, but now the term is used more generally to include sugar-binding proteins from many source regardless of their ability to agglutinate cells. Lectins have been found in plant, viruses, microorganisms and animals. They have been classified according to their sugar-binding specificity. For example, Concanavalin A (Con A), Lens Culinaris Agglutinin (LCA), and Pisum Sativum Agglutinin (PSA) have specificity for glucose; Con A, LCA, Narcissus Pseudonarcissus (NPA), and PSA have specificity for mannose. Most lectins are multimeric, consisting of non-covalently associated subunits, can be homologous identical units or heterologous different subunits. It is this multimeric structure that gives lectins the ability to agglutinate cells or form aggregates with glycoconjugates in a manner similar to the antigen-antibody complex. Although most lectins can agglutinate some cell types, cellular agglutination is not a prerequisite. Some lectins can bind to cells, but would not cause agglutination. Another important characteristics of some lectins is their ability to induce mitosis in cells which are normally not dividing. Con A and PHA-L have been used by immunologists as mitogens to stimulate T cell blastogenesis. Lectins are also used as tools in biochemistry, cell biology, and immunology, and have also been used for diagnostic and therapeutic purposes in cancer research [10-13]. The reversible bindings with free sugars or with sugar residues of polysaccharides, glycoproteins, or glycolipids behave like the antibody and antigen interaction, and are used for glycoprotein purification, oligosaccharide analysis, and cell identification or enrichment. Tumor cells have abnormal expression of lectins. The level of cell surface lectins increases after normal cells are transformed by oncogenes. Tumor cells exhibiting metastatic propensity have higher levels of surface lectins. The tumor cell surface lectins might be involved in cell-cell adhesion, cell attachment to substratum, anchorage-independent growth, and blood-borne metastasis. Malignant transformation is associated with various and complex alterations in the glycosylation process. The lectin-cell surface carbohydrate interactions participate in the process of metastasis. Cell surface carbohydrates affect tumor cell interactions with normal cells or with the extracellular matrix during metastatic spread and growth that is mediated by tumor cell carbohydrates and their binding proteins known as endogenous lectins. Cell glycosylation depends on the expression and function of various glycosyltransferases and glycosidases. Changes of these enzymes in malignant transformation indicate that cell surface carbohydrates affect the metastatic behavior of tumor cells. Some of these changes might provide a selective advantage for tumor cells during their progression to more invasive and metastatic phenotypes. Lectins have been used as tools for diagnosis and therapeutics, and their anti-hepatoma effect involving autophagy will be discussed below.

### Autophagy and cancer

Autophagy is an evolutionarily conserved catabolic process that degrades long-lived proteins, organelles, and bulk cytoplasm [14-16]. The word "autophagy" is derived from the Greek and means to eat (phagy) oneself (auto). Two types of autophagy with dynamic rearrangement of the sequestering membrane in eukaryotic cells are identified: microautophagy and macroautophagy. They differ with respect to the pathway by which cytoplasmic materials are delivered to the lysosome, but share in common the final steps of lysosomal degradation of the cargo with eventual recycling of the degraded materials. Microautophagy is the engulfment of cytoplasm directly at the lysosomal surface while macroautophagy involves the formation of the cytosolic double-membrane vesicles that sequester portions of the cytoplasm or organelles. Fusion of the autophagosome vesicle with the lysosome results in the delivery of autophagic body into the lumen of the degradative compartment and recycling of the nutrients. The autophagy is usually referred to macroautophagy. It occurs as a cellular response to both extracellular stress conditions such as nutrient starvation, hypoxia, overcrowding, heat, drug treatment, and intracellular stress conditions such as accumulation of damage, superfluous organelles and cytoplasmic components, and reactive oxygen intermediates. In mammals, autophagy participates in many physiological processes, including the response to starvation, cell growth control, anti-aging mechanism, innate immunity, and antigen presentation. Pathologically, the deregulation of autophagy is known to play a role in diseases such as cancer, cardiomyopathy, muscular diseases, and neurodegenerative disorders. Autophagy is a dynamic, multi-step process that can be modulated either positively or negatively [17]. An accumulation of autophagosomes can result from a combination of increased autophagic activity and/or a reduced turnover of autophagosomes. Autophagy induction should be differentiated from autophagic flux, a complete process of autophagy from the delivery of cargo to lysosomes and its subsequent breakdown and recycling. Autophagy induction begins with an initiation of phagophore and sequestering the compartment into the autophagosome. A defect in autophagosome turnover may be due to a block in the fusion with lysosomes or disruption of lysosomal functions that results in increased numbers of autophagosome. In this case, an autophagy is induced, but there is no or limited autophagic flux. The incomplete autophagy with the accumulation of autophagosomes contributes to physiological dysfunction. In contrast, complete autophagy will generally exert a cytoprotective effect.

Apoptosis (self-killing) and autophagy (self-eating) are referred to as programmed cell death type I and type II, respectively. Autophagy constitutes a stress adaptation that avoids cell death, whereas in certain conditions, autophagy will lead to an alternative cellular demise that is called autophagic cell death (or type II cell death). Apoptosis is characterized by caspase activation, DNA fragmentation, cytoplasmic blebbing, and engulfment of the apoptotic cell body by other phagocytes. In contrast, cells that die with an autophagic cell death are capable of digesting their own contents without the aid of engulfing phagocytes, in a process that is usually caspase-independent. The morphological hallmark of autophagic cell death is defined by the presence of autophagosomes and autolysosomes in dying cells. The relationship between apoptosis and autophagy is complex. Autophagy and apoptosis may be triggered by common stimuli, and sometimes results in combined autophagy and apoptosis. In other instances, autophagy and apoptosis develop in a mutually exclusive manner, perhaps as a result of variable thresholds for both processes, or as a result of a cellular decision between the two responses that may be linked to a mutual inhibition of the two phenomena. Furthermore, autophagic cell death occurs under physiological conditions during development and is controlled by similar mechanisms as autophagy in cell survival. The role of autophagy is context dependent, as it participates in cell death under some circumstances and promotes cell survival in others [18]. However, if we consider that autophagy is simply a catabolic process that provides energy and resources to many cellular and biological processes, this dichotomy can be resolved.

Autophagy has been observed in cancer cell lines as a reaction toward a variety of metabolic and therapeutic stresses including interruption of growth factor signaling pathway, activation of mitogen-activated protein kinase signaling, inhibition of proteasomal degradation, the accumulation of intracellular calcium, and endoplasmic reticulum stress. However, it is a reversible phenomenon. When the stress is removed, the autophagic response is down-regulated. This is quite different from other stress fates like apoptosis or necrosis that once triggered, are irreversible. The increased ROS production may be the link between signal transduction events and the enzymatic activation of autophagy genes [16]. The autophagic vesicles form around damaged mitochondria and protein once activated. The double-membrane vesicle fuses with the lysosome to degrade the included contents. When the autophagy persists, the continued depletion of organelles and critical proteins in the cell will lead to a caspase-independent form of cell death. However, for cells undergoing persistent autophagy, hallmarks of apoptosis like caspase activation, necrotic cell death, organellar swelling, and plasma membrane rupture can often be observed. This makes it complex and difficult to characterize which predominant mode of cell death is responsible for a population of tumor cells. Furthermore, different situations have been reported. Apoptosis occurs after inhibition of autophagy. Tumor cell lines use autophagy to recycle essential metabolites such as lipids and amino acids for fuelling the bioenergetic machinery in response to the starvation stress, but the inhibition of autophagy will cause an accelerated cell death with apoptotic characteristics [19]. The inhibition of autophagy that leads to apoptosis may be the result of the failure to adapt to stress. In another situation, autophagy can delay or suppress the apoptosis. The chemotherapy drug, cisplatin can induce apoptosis as an effect of its cytotoxicity. However, it also simultaneously bears autophagy induction as an immediate response as cell exposure to the cisplatin. Inhibition of cisplatin-induced autophagy will block the formation of autophagosomes and enhance cisplatin-induced apoptosis. The switch from autophagy to apoptosis by autophagic inhibitors indicates that autophagy induction was responsible for a pre-apoptotic lag phase for cisplatin. The cisplatin-induced autophagy can be considered as an adaptive response that suppresses and delays apoptosis [20]. In contrast, autophagy is induced when apoptosis is inhibited, and the topoisomerase-2 inhibitor or etoposide would induce autophagic cell death in the absence of BAX and BAK [21]. The removal or functional inhibition of essential proteins from the apoptotic machinery can switch a cellular stress response from the apoptotic default pathway to massive autophagy. Autophagy can be induced by cytotoxic chemotherapy, radiation, or kinase inhibitors that disrupt growth factor signaling used in cancer therapy. The autophagic process can ultimately lead to programmed cell death if the stress imposed is sustained.

### Autophagy emerging as new target for cancer therapy

Autophagy plays a protective role in maintaining energy homeostasis and protein and organelle quality control for normal cells. But for cancer cells, it becomes crucial because cancer cells demand high energy for their continuing uncontrolled proliferation. Autophagy functions are particularly important under metabolic stress condition. Any defects in the autophagy mechanism may lead to the accumulation of cellular damage and generation of genome instability, thus prompting tumor initiation and driving cell-autonomous tumor progression. On the other hand, defects in autophagy will also impair the survival of tumor cells post metabolic stress, resulting in cell death by necrosis, apoptosis, or inflammation. The cytokines generated from inflammation may promote tumor growth and non-autonomous tumor progression. In this sense, autophagy protects tumor cells from metabolic stress-induced damage and allows tumor cells to tolerate and recover from stress. It can be viewed as a tumor suppression mechanism [22]. If we can identify the molecules or pathways of autophagy that are required to suppress the tumorigenesis, and know which human tumor has deficient autophagy, then autophagy can emerge as a new target for cancer therapy.

Although autophagy is supposed to maintain cellular metabolism under starvation, remove damaged organelles under stress, and therefore improve the total survival capacity of cells, there is also the question of how, under certain circumstances, autophagy can lead to cell death. It is not dependent solely on either the cell type or the stimulus. The level of autophagy might determine the final outcome, as moderate induction supports cell survival whereas massive and/or prolonged induction leads to cell death. The progressive destruction of cytoplasmic organelles including mitochondria will make autophagy an effector mechanism of cell death. When a lethal stimulus has reached a threshold which makes cells decide to die, either apoptosis or autophagic cell death will ensue. As apoptosis effectors are abundant, this will be the default pathway in most cases. The autophagic death pathway will be the alternative, especially when the apoptotic pathway is inhibited. Furthermore, the formation of autophagosomes might not be enough, but the formation of autolysosomes is required for autophagic cell death. The lysosomal hydrolases that leak into the cytosol might trigger a lysosomal death pathway. Lysosomes contain catabolic hydrolases that participate in the digestion of autophagic materials in acute cell death by altering the lysosomal-membrane permeabilization, or in tissue invasion after release into the extracellular space. The release of cathepsins, other hydrolases, and ROS after lysosomal-membrane permeabilization can cause mitochondrial outer-membrane permeabilization and activate caspase, resulting in classical apoptosis, caspase-independent apoptosis or necrosis-like death [23].

Cancer displays several altered autophagic processes from the induction, regulation and formation of autophagic vesicles, to the fusion and degradation with lysosomes [24]. Tumor environments contains low levels of oxygen and nutrients. Tumor cells not only use glycolysis, but also increase autophagy to fulfill the high energy demand of proliferation. Several tumor-suppressor genes such as p53, PTEN, DAPK, p27^kip^, TSC1/2, DAPK and cancer-causing oncogenes such as Bcl-2, AKT, PI3K, mTOR are autophagy stimulators or inhibitors, respectively. A monoallelic deletion of *beclin 1 *promotes tumorigenesis, as shown by the *beclin 1*^+/- ^mutant mice, which have a high incidence of spontaneous tumors [25,26]. This suggests that autophagy is a tumor suppressive mechanism, and tumor cells are less sensitive toward autophagic induction compared to normal cells. It is speculated that autophagy can limit tumor inflammation, mitigate genome damage and mutation rate upon metabolic stress, or suppress tumor growth. Furthermore, cancer associated altered lysosomal composition and trafficking might influence the autophagic process.

The pro-apoptotic drugs are currently the main chemotherapeutic agents in cancer therapy, but resistance to apoptosis is natural and is frequently observed involving the activation of the PTEN/PI3K/Akt or NF-κB pathway. The pro-autophagic drugs are an alternative to overcome the apoptosis-resistant cancers, but a question remains as to whether autophagy inhibitors or inducers can be used for future cancer treatment [24,27-29]. Currently no specific autophagy modulators are available, but several compounds developed for other purposes have profound effects on autophagy. Tumor cells have evolved to become apoptosis-resistant and are reliant on autophagy to survive the metabolic stress. Since many current cancer therapies (angiogenesis and growth factor and receptor inhibitors) impose metabolic stress, tumor cells may become more vulnerable due to their high energy consumption and increased glycolysis, so that the autophagy inhibitor may be particularly useful. Tumor cells with defective process of apoptosis and autophagy will fail to tolerate metabolic stress and undergo acute necrotic cell death. The necrotic cell death will further stimulate inflammation through the release of high-mobility group B1 (HMGB1), which will then result in activation of the innate immune system and a wound-healing response to become therapeutically useful.

Several chemotherapeutic agents such as arsenic trioxide, tamoxifen, resveratrol, IFN-γ, and radiation have been shown to trigger autophagic cell death in various cancer cell lines, although most of the cell lines are apoptosis-defective. Therefore, even though autophagy primarily serves as a pro-survival response in normal cell homeostasis, it might be able to signal cell death in tumor cells. Drugs or reagents that specifically trigger autophagic cell death should be well-tolerated due to highly specific killing of cancer cells. Furthermore, it can also circumvent apoptosis-resistance. Nevertheless, we are just at the beginning of understanding the complex interplay of autophagy and cancer, as well as the tumor-microenvironment interaction and its interface with the immune system. Therefore, to be able to distinguish between the survival-supporting and death-prompting roles of autophagy becomes increasingly important. The elucidation of the signaling pathways that confer specificity to the autophagic response downstream of different stimuli will help us to select the specific and therapeutic drugs or agents for cancer therapy.

### Con A-induced autophagy

Con A can bind to the mannose moiety of cell surface glycoproteins specifically because the methyl-αD-mannopyranoside can block this interaction. This binding will induce growth inhibition and cell death in a dose- and time-dependent manner. For tumor cell lines, a high dose is cytotoxic whereas a low dose is cytostatic. The Con A sensitivity varies among different cell lines: the IC_50 _of Con A for HepG2, CT-26, ML-1, and Huh-7 are 5, 10, 10, and 20 μg/ml, respectively. But, for lymphocytes, Con A is a T-cell mitogen at doses of 1–10 μg/ml, while a higher dose is still cytotoxic. Lymphocytes are more sensitive to Con A than tumor cell lines probably due to their high content in mannose- or glucose-containing moiety on the cell membrane. It was found that bound Con A on the cell membrane was internalized and accumulated primarily onto the mitochondria as early as 1 h post treatment, and gradually increased mitochondria membrane permeability change. Some of the endocytosized Con A was then degraded in the lysosome. The increased mitochondria membrane permeability would then lead to the treated cell death with Annexin V-positive, but no DNA ladder or typical apoptosis was observed. Instead, cell death proceeded in an autophagic format. The autophagic pathway characteristics including LC3-II formation, double-layer vesicles, BNIP3 induction, and acidic vesicular organelle formation were observed after Con A treatment. The phosphorylated AKT was also down-regulated, indicating that the growth signal of AKT was altered after Con A treatment. With the electronic microscopic observation, a double-layer vesicle and many autophagosomes were detected on the Con A-treated ML-1 cells. The lysosomal activity as detected by acridine orange staining was increased, and the long stable COX-IV was decreased, indicating the increasing activity of lysosomes. However, the autophagic molecule, beclin-1 was not found to increase. In the presence of autophagy inhibitor 3-MA, a class III-PI3K inhibitor which inhibits the pre-autophagosome formation, the induction of LC3-I and LC3-II formation and the autophagic cell death were partially inhibited. However, the pan-caspase inhibitor zVAD-fmk had no effect on Con A-induced cell death [30].

Using siRNA for beclin-1, ATG 5 or LC3, the LC3-II conversion induced by Con A was slightly inhibited by beclin-1 siRNA, but not by ATG 5 siRNA. Although the Con A-induced cell death was not affected by either beclin-1 or ATG 5 siRNA, the Con A-induced LC3-II conversion and cell death were blocked by LC3 siRNA, suggesting that LC3 plays a major role in Con A-induced cell death. The siRNA for BNIP3 inhibited both the LC3-II conversion and cell death. On the other hand, the LC3 siRNA did not affect the BNIP3 induction, indicating that the mitochondria BNIP3 was upstream of LC3-II. Furthermore, the siRNA for AIF slightly inhibited the Con A-induced ML-1 cell death, indicating that the AIF nuclear translocation only partially contributed to the Con A-induced ML-1 cell death. BNIP3, an adenovirus E1B 19-kDa-interacting protein with cell death-inducing property, was detected earlier than the formation of LC3-II. A major function of BNIP3 is to determine the on/off state of the mitochondria permeability transition pore. It was therefore concluded that the Con A-induction is not a typical or global autophagic cell death, and only involves the BNIP3-mediated mitochondria autophagy [30].

The Con A-induced autophagy is an autophagic flux. Con A causes a LC3-II conversion on ML-1 cells and HepG2 cells in a time-dependent manner. Using the GFP-LC3 processing to monitor the delivery of autophagosomal membranes, it was found that the GFP-LC3 was degraded in the lysosome to generate free GFP after Con A treatment. There is co-localization of LC3 and LAMP-1, and the level of LC3-II was increased in the presence of lysosomal protease inhibitors (pepstain and E64D). This suggests that Con A causes autophagic flux on hepatocytes, and a sustained autophagy will lead to cell death.

### The anti-hepatoma effect of Con A

The current treatment for hepatoma is not satisfactory, the major drawback being the common relapse after surgery or chemotherapy treatment. Several reasons can explain the deficiency of the hepatoma therapy. In relation to the anti-tumor immunity in the host, the liver can be considered as an immunologic organ with unique characteristics of immune cells especially with respect to regulation. In the past, the study of immunotherapy to cancer generally used tumors grown on the mouse back that are more easily handled and measured. However, this site is not clinically relevant, especially for liver cancer. The liver is a special organ, with an immunoregulation system which is distinct from that of either spleen or lymph nodes. We therefore need an *in situ *hepatoma model for the evaluation of any anti-tumor reagent, and so a transplantation hepatoma model was accordingly created [31,32]. The transplantable hepatoma cells were injected into the spleen of the syngenic mice, whereupon the tumor cells first colonized in the spleen and then continue their migration into the liver to form various tumor nodules. The pattern and kinetics of the tumor growth are predictable, for example, a transplantation of 1 × 10^6 ^ML-1 cells will generate tumor nodules in the liver, reaching 150–200 tumor nodules of varying sizes (<1 mm, 1–4 mm, >4 mm in diameter) at three weeks post transplantation. The tumor nodule growth is time-dependent, and the hepatoma-bearing mice will die at around day 40 due to overgrowth of tumors. Many variables can be manipulated, for example, the number of hepatoma cells inoculated can be adjusted for different sizes of tumor nodules or survival of the tumor-bearing mice. The treatment can be commenced at any time depending on the tumor load.

Con A is known to be a T cell mitogen and has been shown to induce hepatitis in mice through the triggering of NK T cells and subsequent activation of CD4^+ ^T cells [33,34]. Using our *in situ *hepatoma model, Con A (7.5 mg/kg body weight, twice at three-day interval) administered at one week post transplantation will significantly decrease 150 tumor nodules in the control mice to 40 tumor nodules in the Con A-treated group at 30 days post tumor injection. Around 30 – 40% of the mice were tumor-free. In the survival experiment, the survival of the hepatoma-bearing mice was prolonged from 40 to 70 days after Con A treatment, while 20–30% of the mice were cured. Because repeated injections will desensitize the Con A effect and will generate the anti-Con A antibody, it was given only twice at day 7 and 10, and as a result, the residue tumors would continue growing and kill the non-tumor-free mice. When the dose of Con A and the number of injections were increased, for example to 20 mg/kg and 4 times, the liver tumor nodules could be completely eradicated. The Con A-activated lymphocytes would infiltrate into the liver to kill the hepatoma cells. However, there was no apparent hepatocyte damage at the sub-optimal dose used of 7.5 mg/kg, and the serum alanine transaminase was not elevated during the treatment course. Con A seems to be less cytotoxic to normal hepatocytes in tumor-bearing mice than in naïve mice, probably because the tumor cells sequester more Con A than normal hepatocytes. Indeed, most of the infiltrating lymphocytes were found to be present around the liver tumor nodule post Con A injection with histological tissue staining observation. The therapeutic effect of Con A can be extended to a large tumor burden. For mice that have hepatoma growth for 2 or 3 weeks, Con A only partially inhibited the tumor nodule growth and prolong the mouse survival. The Con A therapeutic effect decreased along with the increased tumor load. The cells participating in this inhibition were further demonstrated to be CD8^+ ^T as indicated by the *in vivo *depletion of CD4^+ ^or CD8^+ ^T cells. Depletion of CD8^+ ^T cells blocked the anti-tumor effect of Con A whereas depletion of CD4^+ ^T cells also partially affected the Con A anti-tumor activity. However, in the control group, the CD4^+ ^T depletion could partially inhibit the tumor formation in the PBS-treated group, indicating that some regulatory CD4^+ ^T might also participate in the anti-hepatoma activity of the liver. Furthermore, the *in vivo *direct effect of Con A is demonstrated in severe combined immune deficiency (SCID) mice. Con A at a high dose of 20 mg/kg can partially inhibit the liver tumor formation in SCID mice at 21 days post tumor inoculation. This indicates that Con A has a direct inhibitory effect on liver tumor nodule formation independently of lymphocyte activation. Interestingly, during the eradication of established hepatoma in the liver, the ML-1 tumor-specific immunities were established and could prevent the next tumor formation. The tumor-free mice after Con A treatment were inoculated subcutaneously with either ML-1 or CT-26 cells dorsally, and the ML-1 cells could no longer grow in ML-1-sensitized mice compared with naïve mice, but CT-26 tumor cells did grow. This suggests that during the Con A-induced eradication of hepatoma, an immune memory was generated and can lead to resistance to further challenge of the same tumor [30].

How the Con A exerts its anti-hepatoma effect is intriguing. The liver, in its function as a filtering organ, can trap blood-born foreign substances, and its anatomic location and unique blood circulation makes it a good site to concentrate and bind Con A. In our *in situ *hepatoma model, Con A trapped in the liver would preferentially bind to hepatoma cells through its specific binding to the high content of mannose residue on tumor cell membranes. We reasoned that the Con A which accumulated in the liver nodules not only directly induces hepatoma cells to undergo autophagic cell death, but also activates and recruits blood-born lymphocytes into the liver. This hepatic inflammation subsequently induces the adaptive immune response against the tumor and leads to liver tumor regression. The Kupffer, NK, NKT, CD4^+ ^T, and CD8^+ ^T cells are activated, but CD8^+ ^T cells are the major effector cells to kill the tumor cells, whereas the CD4^+ ^T cells have an effector function as well as a regulatory function. Initially, the Con A effect is not tumor cell-specific, but as hepatic inflammation proceeds following Con A-mediated destruction and activation, tumor antigens of the hepatoma cells will be processed and presented to the tumor antigen-specific CD4^+ ^T and CD8^+ ^T cells. Tumor-specific immunity is established thereafter. Either professional macrophages or non-professional epithelial cells can phagocytose the dying autophagic cells [35]. It would be interesting to know how the liver Kupffer cells or dendritic cells handle the autophagic hepatoma cells exogenously and present hepatoma cell antigen to CD4^+ ^T and CD8^+ ^T cells in both MHC class II and class I-restricted mechanisms. Con A can stimulate macrophages to up-regulate the TLRs and enhance the cytokine production [36]. The lymphocytes/monocytes are found to be more sensitive to Con A than tumor cells due to their high content of mannose-containing moiety on the cell membrane. Con A at a dose of 1–10 μg/ml is mitogenic, but becomes cytotoxic at dose higher than 40 μg/ml. T cells are reported to undergo autophagy for their death, survival and proliferation [37,38]. Con A can also induce the LC3-II conversion in lymphocytes. The signal pathways are apparently different because the outcomes (mitogenic or autophagic) are distinct. Therefore, the integration or cross-regulation for Con A on different cells or different dosages needs further investigation, and the effect of Con A on the immune system also needs re-evaluation. The autophagic vesicle has recently been shown to participate in the antigen delivery for MHC class II antigen presentation of the cytoplasmic proteins [39,40].

Alternatively, the anti-tumor effect of Con A may be mediated by the NK cells because Miyagi T *et al*. reported that Con A can activate the intrahepatic innate immune cells to provoke an antitumor effect in a NK cell- and IFN-γ-dependent manner in a CT-26 hepatic metastasis model [41]. This observation is distinct from ours, and the dose of Con A used and the immune mechanism involved are different. A non-hepatocytotoxic dose of Con A (2 mg/kg) was used for the purpose of avoiding liver damage, the intra-hepatic NK cells were activated, and the innate immunity with IFN-γ production participated in the prevention of tumor growth in the liver. In contrast, we used a high dose of Con A (hepato-cytotoxic to hepatocytes in naïve mice) and activated the CD8^+ ^T cells to participate in the inhibition of the established liver tumor. Of course the activation of the NK cells is not excluded in our system.

### Chemoprevention of Con A

Many dietary lectins are resistant to digestion, and were shown to be toxic to animals upon oral ingestion presumably because of the intestinal damage after binding to the cells lining the intestinal mucosa. An appreciable portion of the endocytosed lectin can be transported across the gut wall into the systemic circulation. Lectins may therefore be responsible for reported cases of human intoxication associated with the consumption of inadequately cooked beans [42]. Historically, Mistletoe (the galactoside-specific lectin from *Viscum album*) has been introduced for alternative treatment of cancer for a long time in Europe. Extracts from the plant are used in adjuvant cancer therapy as injections. The interest in using the potential health benefits of bioactive polypeptides and proteins derived from soybeans, including lunasin and lectins has increased. Soybeans have been reported to contain a variety of anticarcinogenic phytochemicals. There has also been increased interest in the potential health benefits of bioactive polypeptides and proteins from soybeans, including lunasin and lectins [43,44]. We have observed that orally-fed Con A also inhibited liver nodule formation in our system, and this is intriguing because it might open a new field for chemopreventive treatment in cancer. Natural Con A-like substances that possess both immunomodulating and autophagy-inducing activities, especially in edible vegetable plants, seeds or health food, can be screened and tested for potential use as chemopreventive agents for cancer.

### Lectin immunotherapy versus tumor antigen-specific cancer vaccine

Cancer immunotherapy with tumor-associated antigens has been debated recently. In 2004, Rosenberg et al. reported a low objective response rate (2.6%) in 440 patients with cancer vaccine trials. The current active immunization with specific antigens has limitations or is not effective, and so needs to be re-evaluated [45,46]. Alternative strategies that can mediate cancer regression in preclinical and clinical models are urgently needed. Cancer vaccines constitute a dynamic process involving the host's immune response. A dynamic balance occurs between the induction and maintenance of host immune response and host/tumor factors that have the potential to diminish these responses. The tumor-specific immunity is considered to be an "autoimmunity" response because most tumor-associated antigens are self antigens. The host immune system is strictly regulated by the immune suppressive modalities to suppress the immune responses to "self antigen". The balance of immune-enhancing versus immune-suppressive factors comes into play in the cancer vaccine therapy. Tumor-induced expansion of regulatory T cells has become an obstacle to successful cancer immunotherapy, as inactivation of T regulatory cells is crucial for a successful immunotherapy [47]. However, it is difficult to achieve the therapeutic effect by a single therapy, even with multiple doses of vaccine to boost the immune response and memory. The combination of therapies is therefore becoming a new paradigm. Several strategies are employed for the combination therapy [48]. For example, vaccine therapy can be enhanced by biological adjuvants such as cytokines, danger signals, or immune inhibitors. Multiple vaccine therapies are employed for the sake of minimizing the toxicity, where prime/boost regimens or virus vector deliveries are used. The tumor cells are altered by drugs or radiation to become more susceptible to T-cell-mediated lysis. Moreover, vaccines initiate a dynamic process, so that anti-tumor effects are potentiated by subsequent therapies, and vaccine therapy can be combined with chemotherapy to be more effective.

To stimulate an anti-tumor immune response, the tumor cells have to be immunogenic. During tumor cell death, apoptosis is an immunologically silent type of cell death whereas necrosis is immunogenic and will stimulate an inflammatory immune response [49,50]. The immune response elicited by tumor cells that spontaneously undergo apoptosis will abort incipient tumors during immunesurveillance. On the other hand, immune responses triggered by treatment-induced tumor immunogenic cell deaths will contribute to the therapeutic efficacy of chemotherapy or radiotherapy. Necrosis is a form of cell death with an unregulated or unscheduled process. The disruption of plasma membrane that is characteristic of necrotic cell death will spill the intracellular proteins that subsequently activate a damage response from the host immune system. The brisk inflammatory response and immune amplification of the damage signal is in contrast to apoptotic cells that are silently removed by macrophages. A rapid loss of cellular membrane potential is a key feature of necrosis. The necrosis can result from mitochondrial depolarization, cellular energy depletion, disturbance of Ca^2+ ^homeostasis, activation of PARP, activation of nonapoptotic protease, generation of ROS, and disruption of plasma membrane. All of these events are inter-related and cross-regulated. Energy failure is the major cause of necrosis. In the cells with deficiencies in the apoptotic mechanism, the cells become susceptible to necrosis induction. The key mediator for the necrosis is poly(ADP-ribose) polymerase, a nuclear protein activated by DNA damage, which can rapidly deplete the cell of NAD^+^, the essential cofactor for aerobic glycolysis. This imposes a transient inhibition of glycolysis and inhibition of ATP production. The bioenergetic crisis that occurs with acute NAD^+ ^and ATP depletion in glycolytic cells is associated with the accumulation of high concentrations of intracellular calcium and ROS, which in turn cooperate to activate calpains, phospholipase A2, and the lysosomes were permeabilized to leak the cathepsins. Proteolysis and lipid peroxidation cause wide-spread membrane permeabilization and irreversible necrotic cell death [51].

### Immune responses in the presence of autophagic cell death

Conventional cancer therapies target to cell death via apoptosis, necrosis, mitotic catastrophe, autophagy, and senescence [52]. Each has its own characteristics, and the nature of the host's immune response to these tumor cell deaths is distinct among different stress conditions. Apoptotic cells are cleared by either professional (macrophages or immature dendritic cells) or non-professional phagocytes (fibroblasts, endothelial, epithelial cells). The exposure of the phosphatidylserine and calreticulin on the surface of the apoptotic cells is recognized by the LDL-receptor-related protein on phagocytes, and this results in the engulfment of the apoptotic cells and the suppression of the pro-inflammatory cytokines [53,54]. In most cases, apoptosis is an immune-silent form of cell death. The TGF-β released from the dying cells or the macrophage that ingested apoptotic cells will suppress the pro-inflammatory cytokine production. For the immature dendritic cells, the IL-12 that is important for the T cell activation will be suppressed, and the dendritic cells become tolergenic. On the other hand, necrosis is always an inflammatory cell death. The HMGB1 is normally a nuclear protein that is passively released in the microenvironment by necrotic cells. Its binding to the RAGE receptor and Toll-like receptor on macrophages or dendritic cells will stimulate the secretion of pro-inflammatory cytokines. HMGB1 is an endogenous immune adjuvant released by necrotic cells that promotes the migration and maturation of dendritic cells, and T cell activation [55,56]. Furthermore, the necrosis-induced recruitment of macrophage and T cells and the subsequent macrophage-associated production of angiogenic and growth factors will further accelerate the chronic inflammation and tumorigenesis.

The role of autophagy that participates in both innate and adaptive immunity has recently begun to be revealed. Autophagy is not only a cellular response to restrict viral infections and replication of intracellular bacteria and parasites, but also participates in the MHC class II presentation [57,58]. The antigen presentation by major histocompatibility complex (MHC) molecules that are subsequently recognized by CD4^+ ^T and CD8^+ ^T lymphocytes is critical to elicit an appropriate immune response. The traditional view of CD8^+ ^T lymphocytes interaction with MHC class I molecules that present endogenous antigens (9–11 amino acids) of cytosolic and nuclear origin is processed by the ubiquitinated proteasome degradation system. Whereas MHC class II antigenic peptides which are products of lysosomal degradation are presented to CD4^+ ^T lymphocytes. The two main protein degradation machineries in eukaryotic cells are in charge of different forms of antigen presentation. Peptides generated by the proteasome are presented on MHC class I products, whereas products of lysosomal degradation are displayed on MHC class II. However, a recent study indicates that MHC II ligands can also be of endogenous origin and that the autophagy is the conduit for intracellular antigen access to MHC II processing during its delivery of the autolysosome to the MHC-class-II-loading compartment (MIIC). This provides a cross-presentation for the endogenous protein to an additional class II presentation to CD4^+ ^T cells. In addition to the participation in the antigen presentation, autophagy also plays a role in the survival and death of T cells. Since CD4^+ ^T cells can be activated with the autophagic delivery of intracellular protein for class II presentation, they will certainly contribute to CD4^+ ^T cell immune surveillance of infected and transformed cells. Self-antigen presentation on MHC class II of both thymic epithelial cells and dendritic cells induces central and peripheral tolerance in the CD4^+ ^T cell compartment [59]. Dendritic cells, NK, and T cells can induce autophagy in the infected cells to fight infections, but at the same time the fate of these effector cells is also determined by their own capacity to perform autophagy. Atg 5 deficiency leads to decreased T and B cell numbers in mice, and the Atg 5 deficient CD4^+ ^and CD8^+ ^T cells fail to undergo efficient proliferation upon T cell receptor stimulation [37]. Th2 polarized CD4^+ ^T cells were reported to be more susceptible to autophagic cell death [38]. Autophagy seems to play a role in T cell maintenance during the steady state and after activation. All these data suggest that autophagy-mediated intracellular antigen presentation not only broadens the MHC class II presentation, and enables the immune surveillance and tolerance induction, but also regulates the T cell survival and death. This provides us an opportunity to manipulate the autophagy to enhance the pathogen clearance as well as modulate the anti-tumor immune responses.

Chemotherapy remains the treatment modality of choice for most advanced cancer while immunotherapy is at the infant stage of cancer treatment. The two are regarded as either unrelated or sometimes antagonistic. However, it has become clear that the chemotherapy-induced tumor cell death process will engage with the anti-tumor immune response, and that the two modalities can be synergistically beneficial for cancer treatment [60-64]. The best strategy of immunotherapy will be the association of both a direct cytotoxic drug effect and an indirect immune-mediated cytotoxic effect. The generation of an immunogenic tumor cell death through the induction of calreticulin, HSP, release of inflammatory mediators, proinflammatory cytokines or HMGB1 will favor the recognition of tumor cell antigens by DC, NK, and T cells. The Con A-induced autophagic cell death with necrosis can initiate a tumor cell-specific immune response, but although Con A can initiate the immune response in a tumor cell in a non-specific manner at the beginning, a tumor-specific immune induction occurs during the eradication of tumor cells, which then leads to a tumor-cell specific response during the later stage. In this sense, Con A is a novel type of endogenous cancer vaccine immunotherapy. The combination effect of direct autophagic induction on target cells and indirect immunomodulating activity on lymphocytes via the mannose/glucose binding to tumor cells will induce an *in situ *inflammatory response and the subsequent anti-tumor response.

### Conclusion: Lectin of Con A is a potential anti-hepatoma agent

**Figure 1:**
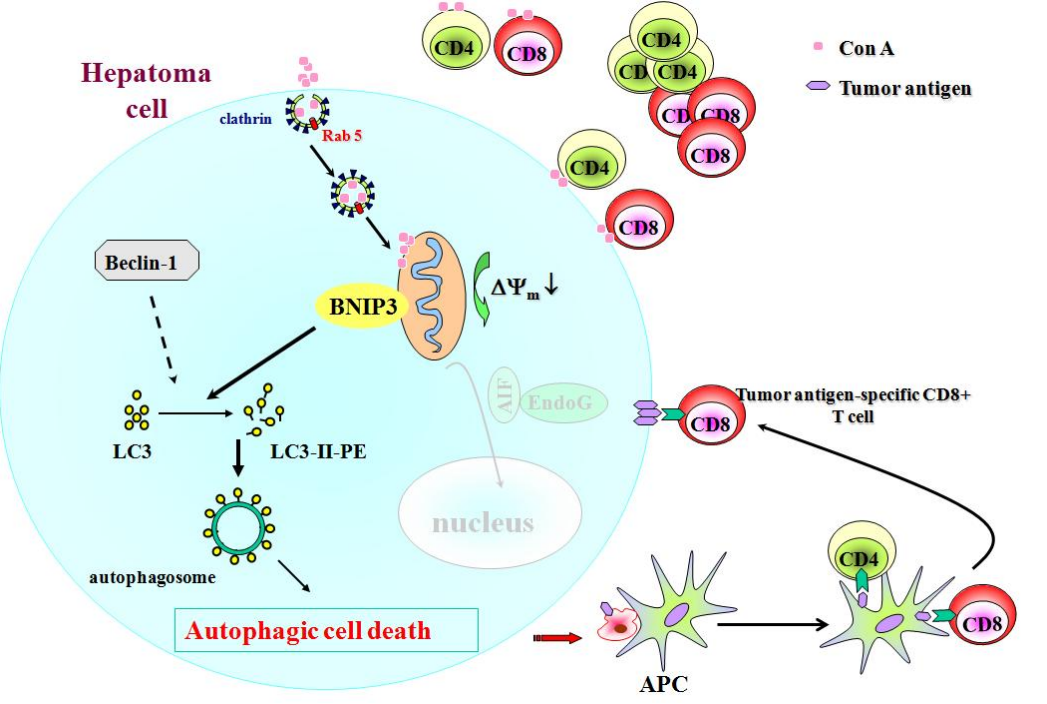
**The molecular mechanism of Con A anti-hepatoma effect**.

Lectins are emerging as bioactive plant proteins to be used in biomedicine, especially for the potential cancer treatment [65-67]. Several lectins have been found to possess anti-cancer properties; they are used as therapeutic agents, preferentially binding to cancer cell membrane, causing cytotoxicity via inducing apoptosis, autophagy, or necrosis, and inhibiting the tumor growth. Furthermore, they could simultaneously activate the immune system, stimulate the cytokine production inside the tumor mass, and the recruited lymphocytes will then participate in the eradication of the tumor. Con A is a prototype of lectins to have this anti-hepatoma effect [68]. We have found that other lectins of Pisum Sativum Agglutinin (PSA) and Lens Culinaris Agglutinin (LCA) from *Pisum sativum *and *Lens culinaris *that specifically bind glucose and mannose also have anti-hepatoma effect. Like Con A, PSA and LCA could also cause hepatoma cell death and are mitogenic to lymphocytes. Both PSA and LCA could significantly inhibit ML-1 liver tumor nodule formation in BALB/c mice. The dual-characteristic of lectins like Con A, springing from their carbohydrate-specific binding, to exhibit both immunomodulating and autophagy-inducing activity, will make them potential candidates for successful anti-hepatoma agents. A cartoon is shown in Figure 1 for its mechanism of anti-hepatoma activity. Moreover, this discovery will open a new area of exploration for natural lectins as anti-cancer compounds.

## Competing interests

The authors declare that they have no competing interests.

## Authors' contributions

HYL conceived of the study, and participated in its design and write the article. CPC carried out the experiments. All authors read and approved the final manuscript.
